# Supplementary material for: Place cells on a maze encode routes rather than destinations
Source: eLife. 2016 Jun 10;5:e15986. doi: 10.7554/eLife.15986 (PMC4942257; doi:10.7554/eLife.15986)
Supplement: Supplementary file 1. — DOI: http://dx.doi.org/10.7554/eLife.15986.017 [file elife-15986-supp1.docx]

| Table 1  *Differential activity in different maze areas, as assessed using three different measures* | | | | | |
| --- | --- | --- | --- | --- | --- |
| Maze Area | | Start Box | Central Stem | Left Stem | Right Stem |
| Active Place Cells | | 176 | 158 | 124 | 115 |
| Differential Activity | Quade Ranked ANCOVA | 83 | 61 | 16 | 14 |
|  |  | 47.2% | 38.6% | 12.9% | 12.2% |
|  | Permutation ANCOVA | 89 | 65 | 26 | 18 |
|  |  | 50.6% | 41.1% | 21.0% | 15.7% |
|  | Generalized Linear Model | 43 | 43 | 37 | 22 |
|  |  | 24.4% | 27.2% | 29.8% | 19.1% |
| Table 1: differential activity table.  Note: percentages are of all place cells assessed for differential activity (i.e. active) in that maze area. | | | | | |

| Table 2  *Frequency of differential cells from Table 1 categorised as coding for each maze route in the start box and central stem of the maze* | | | | | | | | | | | |
| --- | --- | --- | --- | --- | --- | --- | --- | --- | --- | --- | --- |
| Maze Area | | Start Box | | | | | Central Stem | | | | |
| Route | | 1 | 2 | 2&3 | 3 | 4 | 1 | 2 | 2&3 | 3 | 4 |
| Differential Activity | Quade Ranked ANCOVA | 5 | 10 | 2 | 10 | 5 | 4 | 3 | 0 | 8 | 1 |
|  | Permutation ANCOVA | 2 | 12 | 1 | 13 | 4 | 4 | 2 | 0 | 7 | 3 |
|  | Generalized Linear Model | 1 | 3 | 0 | 4 | 1 | 2 | 0 | 0 | 2 | 0 |
| Table 2: start box and central stem differential route coding table.  Note: statistics reported in text are based on the start box and central stem data combined. | | | | | | | | | | | |

| Table 3  *Frequency of differential cells from Table 1 categorised as coding for each maze route in the left and right stems of the maze* | | | | | |
| --- | --- | --- | --- | --- | --- |
| Maze Area | | Left Stem | | Right Stem | |
| Route | | 1 | 2 | 3 | 4 |
| Preferred Route | Quade Ranked ANCOVA | 6 | 10 | 7 | 7 |
|  | Permutation ANCOVA | 10 | 16 | 8 | 10 |
|  | Generalized Linear Model | 17 | 20 | 11 | 11 |
| Table 3: left and right stem differential route coding table.  Note: the numbers in Table 2 are based on post-hoc multiple comparison tests, however, in the left and right stems differential cells are categorised as ‘preferring’ the route where they demonstrated the highest firing rate. | | | | | |

| Table 4  *Results of Chi-square tests on differential cell distributions* | | | | | | | | | | | | |
| --- | --- | --- | --- | --- | --- | --- | --- | --- | --- | --- | --- | --- |
| Statistical Test | Quade Ranked ANCOVA | | | | Permutation ANCOVA | | | | Generalized Linear Model | | | |
| Parameter | N | df | *X^2^* | *p* | N | df | *X^2^* | *p* | N | df | *X^2^* | *p* |
| Overall distribution | 48 | 1 | 16.0 | <.004* | 48 | 1 | 23.0 | <.0001* | 13 | 1 | 8.2 | >.08 |
| Route ( 2 or 3) vs Goal (2 and 3) | 33 | 1 | 25.5 | <.0001* | 35 | 1 | 31.1 | <.0001* | 9 | 1 | 9.0 | <.003* |
| Routes 2&3 vs Routes 1&4 | 48 | 1 | 6.7 | <.015* | 47 | 1 | 9.4 | <.003* | 13 | 1 | 1.9 | >.10 |
| Left and Right Stem Preference | 30 | 1 | 0.5 | >.50 | 44 | 1 | 0.4 | >.50 | 59 | 1 | 0.2 | >.60 |
| Table 4: chi square test results table.  * statistically significant at the .05 level | | | | | | | | | | | | |
